# Research Note: Effects of preincubation and higher initiating incubation temperature of long-term stored hatching eggs on hatchability and day-old chick and yolk sac weight

**DOI:** 10.1016/j.psj.2021.101293

**Published:** 2021-05-27

**Authors:** Martina Pesanova Tesarova, Marketa Skoupa, Marian Foltyn, Zdenek Tvrdon, Martina Lichovnikova

**Affiliations:** ⁎ Department of Animal Breeding, Faculty of AgriSciences, Mendel University in Brno, Brno, Czech Republic; † Výkrm Třebíč, s.r.o., Kutná Hora, Czech Republic

**Keywords:** long-term egg storage, hatchability, preincubation, embryonic mortality, yolk sac

## Abstract

We studied the effect of increased initial incubation temperature and repeated preincubation of 35-d stored eggs from 46-week-old Ross 308 parental stock on the hatchability and day-old chick and yolk sac weight. Two different temperatures were applied during the first 36 h and they were combined with 4 preincubation treatments during storage. One half of the hatching eggs (2,400) were incubated for the first 36 h at an incubation temperature of 38.3°C, and the second half were incubated at a higher temperature of 39.2°C. Four different preincubations were applied; none, once at the 7th d of hatching egg storage, twice at the 7th and 12th d of storage and 3 times at the 7th, 12th and 19th d of storage. Both preincubation and increased temperature had negative effects on hatchability (*P* < 0.001). The interaction between these 2 factors was also significant (*P* < 0.05). These 2 factors also negatively affected early and late embryonic mortality (*P* < 0.001). However, middle embryonic mortality was not influenced. Live weight, weight of residual yolk sac, and yolk sac proportion were not affected by repeated preincubation nor by increased temperature over the first 36 h of incubation (*P* > 0.05). A higher initial temperature decreased chick yolk free body mass (*P* < 0.05). Although neither increased initial temperature in the setter nor repeated preincubation affected one-day-old chick weights, these treatments were not suitable for long-term stored eggs because of decreased hatchability and impairment of one day chick quality expressed as yolk free body mass.

## INTRODUCTION

For many reasons, commercial hatcheries may have to store hatching eggs for an extended period of time before incubation; to synchronize the time of hatching, to limit production depending on the market demand or to assemble a sufficiently large quantity of parental stock hatching eggs (Fasenko, 2007; Gonzalez-Redondo, 2010). Sometimes, long-term storage can extent to more than 25 or even 35 d (Gonzalez-Redondo, 2010). Nevertheless, at the stock parental age of 45 wk, egg storage for 7 d is already associated with a decline in hatchability and chick quality (Tona et al., 2004). Preincubation (warming of hatching eggs before storage) has been a suggested as a method to reduce the negative effect of long-term storage. Some previously published studies indicated that preincubation was associated with improvements in hatchability of chickens (Ebeid et al., 2017). However, Reijrink et al. (2009) suggested that the effect of preincubation on hatchability and chick quality may be influenced by storage time, embryonic development at egg collection and the duration of prestorage incubation. Longer periods of storage cause a further delay in the initiation of development after placement into an incubator (Arora and Kosin, 1966); each day of additional holding produced, on average, a delay of 1 h in development. On the other hand, higher incubation temperatures accelerate embryo development, reflected by heat production (Lourens, et al., 2007). The optimal incubation temperature for wild hens' eggs covers a wide range, ranging from 33°C to 39°C, while a narrower range (37°C to 38°C) is considered optimal for domestic poultry (Visschedijk, 1991). In the study of Sgavioli et al. (2016), incubating eggs at 39°C compromised body weight without yolk and heart development of chicks but hatchability was not influenced (*P* > 0.05). However, when French (2000) applied a temperature of 38.5°C for 5 d to turkey eggs, detrimental (*P* < 0.05) effects on hatchability were observed. Overheating applied during the 2nd or 3rd quarter of the incubation period, an increased incidence of malpositioned embryos were also noted in turkey (French, 2000).

As storage of hatching eggs prolongs the length of the incubation and higher temperatures increase embryo development, the aim of this study was to evaluate the effect of a slightly higher temperature applied at the beginning of incubation, and repeated preincubation of 35 d stored hatching eggs, on hatchability and day-old chick and yolk sac weight.

## MATERIALS AND METHODS

The study was carried out in the hatchery of Vykrm Trebic s.r.o. A total of 4,800 hybrid combination Ross 308 hatching eggs were used, from 46-week-old parental stock.

### Treatments

Hatching eggs were divided into 8 experimental groups, each of 600 eggs. All 4,800 eggs were collected on the same day from the same parental stock and then randomized among the treatments. These eggs were sat in 10 trays, 60 eggs per tray, where one tray represents one replication. Each treatment therefore had ten replications. During storage each treatment had its own trolley, where all trays were put together. All eggs were stored for 35 d (storage room temperature was 14°C, 80% RH, total capacity 1,036,800 eggs). Two different temperatures were applied during first 36 h of incubation and these were combined with 4 preincubation treatments during storage.

Half of the hatching eggs (2,400) were incubated for the first 36 h in the setter at an incubation temperature of 38.3°C (NORM, 100.9°F), which is normally applied in the hatchery. The second half of hatching eggs were incubated for the first 36 h at an increased temperature of 39.2°C (INCR, 102.5°F) to stimulate metabolism of embryos of long-stored eggs. Four different preincubations were applied; none (NORM-0, INCR-0), once at the 7th d of hatching egg storage (NORM-1, INCR-1), twice at the 7th and 12th d of storage (NORM-2, INCR-2), and 3 times at the 7th, 12th, and 19th d of storage (NORM-3, INCR-3). Preincubation was applied in special Re-Store (Petersime NV, Zulte, Olsene, Belgium) machines for 1 h, and the eggshell temperature during this period was 35 °C (95°F). During preincubation the Re-Store were full of its capacity, 57,600 eggs. Temperature was monitored by OvoScan (Petersime NV, Zulte, Olsene, Belgium) on 3 positions of the trolley; upper, middle and lower trays, in total eggshell temperature of twelve eggs was monitored each time.

### Egg Incubation Condition

Storage eggs was set for 35 d in a Biostreamer 24S incubator (Petersime, NV, Zulte, Olsene, Belgium). Half of the hatching eggs (NORM-0, NORM-1, NORM-2, NORM-3, in total, 2400) were incubated at a temperature of 38.3°C. The second half of the hatching eggs (INCR-0, INCR -1, INCR -2, INCR -3, total 2400) were incubated for the first 36 h at a 1°C higher temperature and after that the temperature was returned to NORM for hatching eggs. The temperature in the setters was controlled by OvoScan (Petersime NV, Zulte, Olsene, Belgium) until d 18 of incubation. At d 18 of incubation, the eggs were transferred to the Biostreamer 8H hatcher, where they were incubated until the handling, 518 h after setting, according to the standard program specified by the technology manufacturer. The incubation was carried out at full capacity of the incubator; the setter 115,200 eggs and the hatcher 38,400 eggs.

### Hatchability and One-Day-Old Chick and Yolk Sac Weight

At d 7 of incubation, all hatching eggs were candled, and clear eggs were opened to macroscopically determine embryonic mortality and fertility; blastoderms and blastodics were differentiated. On hatching day, live hatched chicks were counted per basket. All unhatched eggs were opened to determine the stage of embryo mortality; early stage embryonic mortality to 9 d of incubation (black eye visible, embryo without feathers), middle stage embryonic mortality 10 to 17 d of incubation (small embryo with feathers), and late stage embryonic mortality 18 to 21 d (full grown embryo with yolk out or full grown embryo dead or alive with yolk subtracted, Reijrink et al., 2009). The incidence of malposition (head between thighs, head in the small end of egg, head under left wing, head not directed toward air cell, feet over head, beak above right wing) and malformation (exposed brain, without eye(s), 4 legs, deformed beak, no upper beak, deformed twisted leg) were also noted (Butcher and Nilipour, 2018). Because fertility was determined macroscopically, it is possible that an embryo that died during storage was classified as an infertile egg, therefore hatchability and embryonic mortality were calculated as a percentage of set eggs, where egg origin was the same for all treatments. Twelve randomly selected chicks per treatment were weighed and, after euthanasia by decapitation, residual yolk was weighed.

### Statistical Analysis

Observed characteristics were expressed by means. The results for the incubation variables were analyzed by ANOVA with a general linear model procedure (Unistat 5.1 software, UNISTAT Ltd, London, England). Between-tray variation (residual) was the source of the error term; temperature for the first 36 h and the preincubation number were the main effects. Mean differences were tested using the Duncan's multiple range test for all characteristics connected with hatchability and Tukey-HSD test for characteristics connected one-day-old chick and yolk sac weight to evaluate differences among means at *P* ≤ 0.05.

## RESULTS AND DISCUSSION

The effects of increased incubation temperature during the first 36 h and preincubation of 35 d stored eggs are shown in Table 1. Both, preincubation and increased temperature had negative effects on hatchability (*P* < 0.001). Interaction between these 2 factors was also significant (*P* < 0.05). Hatchability was the highest in NORM-0 and the lowest in INCR-3 (*P* < 0.05). These 2 factors also had negative effects on early (*P* < 0.001) and late embryonic mortality (preincubation *P* < 0.05, temperature *P* < 0.01). The highest early embryonic mortality was found in INCR-3 (59.9%, *P* < 0.05). However, middle embryonic mortality and incidence of malformations were not affected. The lowest incidence of malposition was observed in treatments NORM-0 and INCR-3. This parameter was affected by both preincubation (*P* < 0.001) and temperature (*P* < 0.01), including interaction of these factors (*P* < 0.001).

**Table 1:** Effects of repeated preincubation and increased hatching temperature on hatchability and embryonic mortality.

Treatment	Hatchability of set eggs (%)	Apparent fertility (%)	Embryonic mortality (%)			Malpositions (%)	Malformations (%)
			Early 1–9 d	Middle 10–17 d	Late 18–21 d		
NORM-0	61.5^a^	94.3^a^	25.8^d^	0.67^a^	1.17^c^	4.67^c^	0.51^a^
INCR-0	41.4^bc^	92.4^ab^	37.4^c^	0.34^a^	3.41^abc^	8.65^b^	1.20^a^
NORM-1	36.6^cd^	91.0^abc^	41.6^c^	0.87^a^	4.05^ab^	7.32^bc^	0.52^a^
INCR -1	23.3^e^	94.1^a^	50.3^b^	1.72^a^	4.13^ab^	13.13^a^	1.56^a^
NORM-2	42.6^b^	91.2^abc^	36.8^c^	0.53^a^	1.77^bc^	9.01^b^	0.53^a^
INCR -2	33.6^d^	88.7^c^	38.7^c^	1.70^a^	1.37^bc^	12.27^a^	1.01^a^
NORM-3	33.9^cd^	83.8^d^	39.0^c^	0.86^a^	0.68^c^	8.53^b^	0.85^a^
INCR -3	16.6^f^	89.0^bc^	59.9^a^	1.37^a^	5.64^a^	4.99^c^	0.51^a^
P level							
Preincubation	<0.001	<0.001	<0.001	NS	<0.05	<0.001	NS
Temperature	<0.001	NS	<0.001	NS	<0.01	<0.01	NS
Preinc x temper	<0.05	<0.05	<0.001	NS	<0.05	<0.001	NS

^a,b^Means in the same column designated by different letters are significantly different (*P* ˂ 0.05).

Abbreviation: NS, nonsignificant.

The highest hatchability of set eggs was found in NORM-0 (61.5%). At the age of parent stock 46 wk, the hatchability 61.5% is poor in comparison with 84.0% hatchability of set eggs stored up to 7 d at parent stock age 43 to 48 wk (Damaziak et al., 2021). On the other hand, Yassin et al. (2008) in the field study reported similar or even lower hatchability; average estimated hatchability at 25 wk of age was 66% and decreased to 50% at 65 wk of age. Extending egg storage from 4 d to 14 d decreased the hatchability of set/fertile eggs and increased embryonic mortality in Egyptian local cross Inshas (*P* < 0.05, Ebeid et al., 2017). Arora and Kosin (1966) reported that after 35 d turkey egg storage, early embryonic mortalities after 3 d of incubation were 88.5 and 89.4%, and the detrimental effect of storage was evident even after 7 d. Tona et al. (2004) noted a decrease in hatchability after 7 d storage at the parental stock age of 45 wk; the age of the parental stock used in our study was 46 wk.

Gonzalez-Redondo (2010) recorded a decrease in hatchability of red-legged partridge after 35 d of egg storage, which were turned 2 times per day at regular intervals (*P* < 0.01). However, after storage for 0, 7, 14, 21 and 28 d, he did not observe any difference (*P* > 0.05). A hatchability of 61.5% after 35 d of storage is quite high; Sittmann et al. (1970) reported hatchability from 4.6 to 8.8% of chicken eggs stored 31 to 36 d at 15.5°C however without turning. Arora and Kosin (1966) hypothesize that long and intensive genetic improvement of the chicken has produced a species genotype that makes the embryo more fit to overcome the deleterious effect of prolonged preincubation storage.

No effect of the factors was observed on chicks’ malformations. Arora and Kosin (1966) found an effect of lengthy storage on early embryonic malformations, causing early embryonic mortality; possibly the same situation occurred in our study, resulting in increased early embryonic mortality but without incidence of malformations in later embryonic development. A higher incubation temperature accelerates embryo metabolism; this was the reason a higher temperature was applied in 35 d stored eggs. Increased temperature during the first 36 h of incubation, from 38.3 to 39.2°C, had a negative effect on hatchability, and early and late embryonic mortality (*P* < 0.001). Sgavioli et al. (2016) conducted a similar experiment with fresh layer eggs, using a standard temperature of 37.5°C and an increased temperature of 39.0°C for 18 d. Hatchability and embryo mortality were not significantly affected by incubation temperature in their experiment despite the difference 8.43% (89.89% vs. 81.46%; n = 2 trays of 90 eggs, 180 eggs per treatment, using Tukey test). It seems that adverse effects in our experiment were associated with the long storage period. On the other hand, Narinc et al. (2016) applied a higher temperature (39.6°C) for 6 h/d until 8 d of incubation on broiler eggs, and observed a significantly lower hatchability in fresh eggs (90.32% vs. 89.26%; 200 eggs per treatment, using Duncan's multiple comparison test).

Reijrink et al. (2009) did not observe any effect of preincubation on the hatchability of eggs stored for 3, 5 or 8 d, but in eggs stored for 11 d, preincubation for 4.5 h led to higher hatchability (*P* < 0.05), but incidence of second grade chicks was slightly higher in the treatment with preincubation (1.0% vs. 2.3%). However, in eggs stored for 12 d, preincubation for 6 h led to lower hatchability (*P* < 0.05). Ebeid et al. (2017) reported a positive effect of preincubation for 4, 6 or 8 h at 37.5°C on eggs from 28 wk parental stock stored for 14 d, but from 50 wk parental stock, a positive effect was observed only with 8 h of treatment.

Table 2 shows the results of one-day-old chick and yolk sac weight. Live weight, weight of residual yolk sac and yolk sac proportion were neither affected by repeated preincubation nor by increased temperature at the first 36 h of incubation (*P* > 0.05). Differences in live body weight and yolk free body mass were observed only between NORM-0 (46.8g) and INCR-0 (42.5g, *P* < 0.05) and NORM-0 (40.2g) vs. INCR-0 (36.7g, *P* < 0.05) respectively. Yolk-free body mass is a better indicator of broiler development than body weight, because the latter included the weight of the residual, not yet metabolized yolk (Wolanski et al., 2004). In the present study, the quality of the chicks derived from eggs incubated at a high temperature was worse than those incubated at the standard temperature, as shown by their lower yolk-free body mass (*P* < 0.05). On the other hand, Wineland et al. (2006), who used incubation temperatures from 36 to 39°C did not see any differences in yolk weight. Anyway they concluded, that data indicated that temperatures greater than 37°C slowed yolk-free body growth, but also spared residual yolk. Thus, although chicks may appear to be heavy at hatching at high temperatures, more residual yolk mass was responsible for the difference and not tissue mass. Sgavioli et al. (2016) reported that eggs incubated at a high temperature exhibited a greater loss of egg mass than those incubated at the control temperature, which could explain the lower live weight of day old chicks in our experiment, in treatment INCR-0.

**Table 2:** Effects of repeated preincubation and increased hatching temperature on the one-day-old chicks and yolk sac weight.

Treatment	Live weight (g)	Yolk sac weight (g)	Yolk sac proportion (%)	Yolk free body mass (g)
NORM-0	46.8^a^	6.61^a^	14.1^a^	40.2^a^
INCR-0	42.5^b^	5.78 ^a^	13.5^a^	36.7^b^
NORM-1	44.8^ab^	6.91^a^	15.4^a^	37.9^ab^
INCR-1	44.9^ab^	6.64^a^	14.8^a^	38.2^ab^
NORM-2	46.3^ab^	6.73 ^a^	14.5^a^	39.5^ab^
INCR-2	44.9^ab^	6.78^a^	15.1^a^	38.1^ab^
NORM-3	45.6^ab^	6.03^a^	13.3^a^	39.6^ab^
INCR-3	45.6^ab^	6.77^a^	14.7^a^	38.8^ab^
*P* level				
Preincubation	NS	NS	NS	NS
Temperature	NS	NS	NS	˂0.05
Preinc x temper	NS	NS	NS	NS

^a,b^Means in the same column designated by different letters are significantly different (*P* ˂ 0.05).

Abbreviation: NS, Nonsignificant.

Although storing hatching eggs for 35 d is not common practice in the hatcheries, neither increased temperature at the beginning of incubation nor preincubation helped improve hatchability. Both factors had a negative effect on hatchability, early and late embryonic mortality and on the incidence of malpositions. An increased initial temperature decreased the quality of one-day-old chick as assessed by yolk free body mass.
